# Gender-related differences in heart failure with preserved ejection fraction

**DOI:** 10.1038/s41598-018-19507-7

**Published:** 2018-01-18

**Authors:** Franz Duca, Caroline Zotter-Tufaro, Andreas A. Kammerlander, Stefan Aschauer, Christina Binder, Julia Mascherbauer, Diana Bonderman

**Affiliations:** Division of Cardiology, Department of Internal Medicine II, Medical University of Vienna, Vienna, Austria

## Abstract

Heart failure with preserved ejection fraction (HFpEF) affects more women than men, suggesting gender to play a major role in disease evolution. However, studies investigating gender differences in HFpEF are limited. In the present study we aimed to describe gender differences in a well-characterized HFpEF cohort. Consecutive HFpEF patients underwent invasive hemodynamic assessment, cardiac magnetic resonance imaging and exercise testing. Study endpoints were: cardiac death, a combined endpoint of HF hospitalization or cardiac death and all-cause death. 260 HFpEF patients were prospectively enrolled. Men were more compromised with regard to exercise capacity and had significantly more co-morbidities. Men had more pronounced pulmonary vascular disease with higher diastolic pressure gradients and a lower right ventricular EF. During follow-up, 9.2% experienced cardiac death, 33.5% the combined endpoint and 17.3% all-cause death. Male gender was independently associated with cardiac death, but neither with the combined endpoint nor with all-cause mortality. We detected clear gender differences in HFpEF patients. Cardiac death was more common among men, but not all-cause death. While men are more prone to develop a right heart phenotype and die from HFpEF, women are more likely to die with HFpEF.

## Introduction

Heart failure (HF) is a highly morbid condition with increasing prevalence, already affecting more than 23 million people worldwide^1^. Approximately 50 percent of all HF patients have a preserved left ventricular (LV) ejection fraction (EF)^2^, a condition that has been termed heart failure with preserved ejection fraction (HFpEF). The complex pathophysiology of HFpEF is still incompletely understood and no therapy is available to improve patient outcomes^3^.

Over the past decade, one of the most robust findings across numerous HFpEF studies was a distinct gender distribution. Namely, women significantly outnumber men, leading to a gender ratio of approximately 2:1 in HFpEF^4,5^. This supports the notion that gender plays a crucial role in this increasingly recognized condition. However, prospective studies investigating gender differences in HFpEF are scarce^6–8^ and were mostly performed within the context of clinical trials, which have the disadvantage that they may not necessarily reflect real-life HFpEF patients. Also, earlier studies did not actively screen for coronary artery disease (CAD) or cardiac amyloidosis. Therefore, relatively little is known about the influence of gender on clinical course and outcome in pure HFpEF cohorts without overlapping cardiac conditions, such as CAD, or mimicking conditions, such as amyloidosis^9,10^.

To shed more light on the influence of gender on the course of HFpEF patients we investigated a well-characterized HFpEF cohort, from a prospective national registry and assessed differences between men and women with regards to clinical, hemodynamic and imaging parameters, as well as outcome data.

## Methods

### Study design

This was a prospective observational study performed at the Division of Cardiology of the *Medical University of Vienna*, a national HFpEF referral center with a high volume HF outpatient clinic, multimodality imaging, and cardiac catheterization laboratories. The present study was conducted in accordance with the *Declaration of Helsinki*, the study protocol was approved by the ethics committee of the *Medical University of Vienna* (EK# 796/2010) and all patients gave written informed consent before enrollment.

### Clinical definitions

HFpEF was diagnosed according to current consensus statements of the European Society of Cardiology^3^ and the American Heart Association^11^.

Inclusion criteria for this study were [1] signs or symptoms of HF, [2] LVEF ≥ 50%, [3] N-terminal prohormone of brain natriuretic peptide (NT-proBNP) > 220 pg/mL, and [4] evidence of LV diastolic dysfunction by transthoracic echocardiography (TTE). LV diastolic dysfunction was assessed via the ratio of early transmitral blood velocity (E) to early diastolic mitral annular velocity (e’). Diastolic dysfunction was diagnosed, if E/e’ was > 15. Patients with E/e’ between 8–15 represented intermediate cases in whom diastolic dysfunction was considered, but could neither be confirmed nor excluded. Diastolic dysfunction was excluded in patients with E/e’ ≤ 8. If diastolic dysfunction and HFpEF were likely after TTE and NT-proBNP assessment, right heart catheterization was performed in order to confirm the diagnosis. The diagnosis was confirmed, if pulmonary artery wedge pressure (PAWP) was >12 mmHg^12^.

Main exclusion criteria were significant CAD, which was assessed by coronary angiography and defined as a visual stenosis over 50% in one of the main vessels and/or over 70% in one of the distal vessels, significant valvular or congenital heart disease, severe chronic obstructive lung disease (COPD) (Global Initiative for Chronic Obstructive Lung Disease grade III or IV) and cardiac amyloidosis. Cardiac amyloidosis was diagnosed in accordance with current recommendations using cardiac magnetic resonance imaging (CMR), ^99^mTc-3,3-diphosphono-1,2-propanodicarboxylic acid scintigraphy, serum and urine immunofixation and if necessary, endomyocardial biopsy^13^.

### Outcomes

Three different endpoints were assessed: [1] cardiac death, [2] cardiac death or HF hospitalization and [3] all-cause death. To ascertain study endpoints, patients were followed via outpatient visits or telephone calls in case of immobility. If an event occurred, local and external medical records as well as conversations with the patients and/or their relatives were used for preparation of endpoint-reports, which were reviewed by a clinical adjudication committee (D.B, S.A, J.M).

Death from right heart failure (RHF) was defined as follows: [1] Presence of right ventricular (RV) dysfunction on TTE and/or CMR. RV dysfunction on echocardiography was defined as tricuspid annular plane systolic excursion (TAPSE) < 16 mm and/or RV fractional area change (FAC) < 35%. Parameters were measured according to current guidelines^14^. RV dysfunction was confirmed on CMR, if RVEF was < 45%^15^. [2] Presence of clinical signs and symptoms of RHF such as dyspnea, peripheral edema, ascites, elevation of liver enzymes or jugular venous distension at time of death. Sudden cardiac death (SCD) was defined as sudden, unexpected death from cardiac arrhythmia (documented on ECG) without prior circulatory failure, or in case of out-of-hospital SCD, death following an unexpected, sudden collapse without pulse or respiration in the absence of an obvious non-cardiac reason.

### Right heart catheterization

A 7 french Swan-Ganz catheter (Baxter, Irvine, CA, USA), inserted via either jugular or femoral access, was used for measurement of invasive hemodynamic parameters. Filling pressures were calculated as the average over eight heart cycles (CathCorLX, Siemens AG, Berlin and Munich, Germany). Hemodynamic parameters of interest were systolic pulmonary artery pressure (sPAP), diastolic pulmonary artery pressure (dPAP), mean pulmonary artery pressure (mPAP), PAWP, right atrial pressure, cardiac index, stroke volume index, diastolic pressure gradient (DPG), and pulmonary vascular resistance (PVR). DPG and PVR were calculated as follows: DPG = dPAP – PAWP, PVR = 80* [(mPAP − PAWP)/cardiac output].

### Cardiac magnetic resonance imaging

Patients without contraindications underwent CMR on a 1.5 Tesla scanner (MAGNETOM Avanto, Siemens Healthcare GmbH, Erlangen, Germany). All CMR studies were performed according to standard protocols including functional and late gadolimium enhancement imaging^16^.

### Transthoracic echocardiography

All patients underwent TTE on high-end scanners (GE Vivid 5 and Vivid 7; GE Healthcare, Wauwatosa, WI, USA). All examinations were performed by board-certified physicians. in accordance with current guidelines^14,17^. Parameters of interest were E/e’, TAPSE, FAC, degree of tricuspid regurgitation and sPAP. Moderate or severe tricuspid regurgitation were considered significant^18^. Furthermore, the ratio between TAPSE and sPAP was assessed in order to characterize RV function with an afterload-independent parameter^19^.

### Exercise capacity

To assess submaximal exercise capacity, the 6-minute walk test was used. Tests were performed according to the guidelines of the American Thoracic Society indoors on a 50 meter track^20^. To account for gender differences the percentage of predicted 6-MWD [(6-MWD/predicted 6-MWD using the Enright formula)*100] was used for analysis.

### Statistical analysis

To account for gender differences, all applicable parameters were indexed to body surface area using the DuBois formula. IBM SPSS version 22 (SPSS Inc., Chicago, IL, USA) was used for statistical analysis. Level of significance was set to P ≤ 0.05. Categorical variables are expressed as numbers and percent. Continuous variables are expressed in median and interquartile ranges (IQR). To assess differences in baseline parameters between male and female HFpEF patients, Mann-Whitney U test or chi-square test were used as appropriate. To assess the effect of gender on outcome, Kaplan-Meier curves (Log rank test), uni- and multivariable Cox regression models were computed for the endpoints cardiac death, cardiac death or HF hospitalization, and all-cause death. After univariable Cox regression, all significant parameters were entered in a multivariable model with stepwise forward selection with a significance level of 0.05 to enter the model and a significance level of 0.1 to stay in the model.

### Data availability

Data will be made available upon request.

## Results

### Study population

Between December 2010 and November 2016, a total of 302 patients were referred. 15 patients were excluded because of significant CAD, 14 because of NT-proBNP ≤ 220 pg/mL and 13 because of cardiac amyloidosis. 260 patients with a definite diagnosis of HFpEF entered the registry.

### Baseline characteristics

Baseline characteristics for the study cohort are displayed in Table 1. Median age of the study population was 73.0 years (IQR: 67.0–77.0) and NT-proBNP was markedly elevated (1169 pg/mL, IQR: 557–2072). Almost two thirds of patients presented with New York Heart Association class ≥ III, co-morbidities such as arterial hypertension, morbid obesity, atrial fibrillation, chronic kidney disease, or diabetes mellitus type II were frequently present. Furthermore, pulmonary hypertension (PH) was frequent (71.9%) among study participants. 59.6% had isolated post-capillary pulmonary hypertension (IpcPH) and 12.3% had combined post- and pre-capillary pulmonary hypertension (CpcPH).

**Table 1:** Baseline characteristics.

Variable	Total HFpEF cohort (n = 260)	Female HFpEF cohort (n = 181)	Male HFpEF cohort (n = 79)	*P* value
Clinical parameters				
Age, years	73.0 (67.0–77.0)	73.0 (67.5–77.0)	72.0 (66.0–77.0)	0.237
Body mass index, kg/m^2^	29.6 (25.5–34.3)	29.3 (24.4–34.9)	30.2 (26.9–33.4)	0.443
**% of 6** **minute walk distance predicted, %**	76.6 (54.4–91.7)	78.1 (58.5–92.4)	66.7 (39.8–91.6)	**0**.**036**
NYHA functional class ≥ III, n (%)	170 (65.4)	122 (67.4)	48 (60.8)	0.201
NT-proBNP, pg/mL	1169 (557–2072)	1150 (524–1991)	1209 (619–2231)	0.300
Combined endpoint, n (%)	87 (33.5)	54 (29.8)	33 (41.8)	0.061
**Cardiac death**, **n** (**%**)	24 (9.2)	11 (6.1)	13 (16.5)	**0**.**008**
**Non-cardiac death**, **n** (**%**)	21 (8.1)	19 (10.5)	2 (2.5)	**0**.**030**
**Co-morbidities**				
**Atrial fibrillation**, **n** (**%**)	154 (59.2)	99 (54.7)	54 (68.4)	**0**.**040**
Non-significant coronary artery disease, n (%)	64 (24.6)	40 (22.1)	24 (30.4)	0.154
Arterial hypertension, n (%)	249 (95.8)	173 (95.6)	76 (96.2)	0.819
Pulmonary hypertension, n (%)	187 (71.9)	127 (70.2)	60 (75.9)	0.985
Isolated post-capillary pulmonary hypertension, n (%)	155 (59.6)	109 (60.2)	46 (58.2)	0.121
Combined post- and pre-capillary pulmonary hypertension, n (%)	32 (12.3)	18 (9.9)	14 (17.7)	0.121
Chronic kidney disease*, n (%)	140 (53.8)	98 (54.1)	42 (53.2)	0.884
Hyperlipidemia, n (%)	143 (55.0)	97 (53.6)	46 (58.2)	0.489
Morbid obesity^†^, n (%)	129 (49.6)	86 (47.5)	43 (54.4)	0.388
Diabetes mellitus type II, n (%)	96 (36.9)	62 (34.3)	34 (43.0)	0.177
**Anemia**, **n** (**%**)	168 (64.6)	110 (60.8)	58 (73.4)	**0**.**050**
**Sleep apnea**, **n** (**%**)	25 (9.6)	9 (5.0)	16 (20.3)	**<0**.**001**
**Smoker** (**former or active**), **n** (**%**)	75 (28.8)	43 (23.8)	32 (40.5)	**0**.**009**
**Chronic obstructive pulmonary disease**, **n** (**%**)	86 (33.1)	49 (27.1)	37 (46.8)	**0**.**002**
**Concomitant medication**				
Beta Blockers, n (%)	196 (75.4)	132 (72.9)	64 (81.0)	0.157
ACE inhibitors, n (%)	81 (31.2)	50 (27.6)	31 (39.2)	0.063
Angiotensin receptor blockers, n (%)	98 (37.7)	74 (40.9)	24 (30.4)	0.108
Calcium channel blockers, n (%)	76 (29.2)	50 (27.6)	26 (32.9)	0.389
Antiarrhythmic agents, n (%)	23 (8.8)	17 (9.4)	6 (7.6)	0.639
Loop diuretics, n (%)	147 (56.5)	99 (54.7)	48 (60.8)	0.364
Thiazide diuretics, n(%)	80 (30.8)	62 (34.3)	18 (22.8)	0.065
Mineralocorticoid receptor antagonist, n (%)	95 (36.5)	62 (34.3)	33 (41.8)	0.247
Antidepressants, n (%)	60 (23.1)	46 (25.4)	14 (17.7)	0.176
Oral anticoagulants, n (%)	161 (61.9)	109 (60.2)	56 (70.9)	0.100
Antiplatelet agents, n (%)	82 (31.5)	60 (33.1)	22 (27.8)	0.398
Statins, n (%)	127 (48.8)	90 (49.7)	37 (46.8)	0.668
**Invasive hemodynamic parameters**				
Mean pulmonary arterial pressure, mmHg	33.0 (27.8–39.3)	33.0 (26.3–39.0)	34.0 (29.0–40.3)	0.784
Right atrial pressure, mmHg	12.0 (8.0–16.0)	12.0 (8.0–15.0)	12.0 (8.5–17.0)	0.625
Pulmonary artery wedge pressure, mmHg	20.0 (16.8–24.0)	20.0 (17.0–24.0)	19.0 (15.0–22.0)	0.059
Stroke volume index, mL/m^2^	38.5 (32.2–45.8)	37.0 (31.2–44.9)	41.0 (32.6 – (47.8)	0.144
Cardiac index, L/min/m^2^	2.7 (2.3–3.0)	2.7 (2.3–3.0)	2.7 (2.3–3.3)	0.370
Pulmonary vascular resistance, dyn·s·cm^−5^	202 (148–282)	204 (147–284)	191 (147–278)	0.659
**Diastolic pressure gradient**, **mmHg**	2.0 (−1.0–5.0)	1.0 (−2–4.5)	3.0 (0.0–6.0)	**0**.**010**
**Cardiac magnetic resonance imaging parameters**				
Left atrial volume index, mL/m^2^	62.0 (45.3–78.0)	60.0 (45.0–76.5)	65.0 (51.0–86.0)	0.456
Right atrial area index, mm/m^2^	14.3 (12.3–18.5)	13.7 (12.2–17.7)	15.8 (12.6–19.9)	0.062
**Left ventricular ejection fraction**, **%**	63.0 (55.0–72.0)	66.0 (55.5–74.0)	58.0 (53.0–66.0)	**0**.**005**
Left ventricular end-diastolic volume index, mL/m^2^	63.4 (53.5–75.0)	63.1 (53.3–74.4)	65.3 (55.1–80.8)	0.372
**Left ventricular mass index**, **g/m**^**2**^	55.5 (48.0–65.3)	53.6 (44.3–63.2)	61.7 (52.5–74.9)	**0**.**002**
**Right ventricular ejection fraction**, **%**	52.0 (45.0–60.0)	55.0 (45.0–63.0)	50.0 (44.0–54.8)	**0**.**006**
**Right ventricular end-diastolic volume index**, **mL/m**^**2**^	76.0 (62.7–90.5)	73.7 (62.0–84.5)	85.0 (66.8–96.1)	**0**.**011**
**Echocardiographic parameters**				
E/e’	13.7 (10.3–18.5)	13.1 (10.4–20.0)	14.1 (10.2–18.0)	0.881
TAPSE, mm	19.0 (16.0–23.0)	19.0 (16.0–22.0)	19.0 (15.8–23.3)	0.838
Systolic pulmonary arterial pressure, mmHg	56.0 (46.0–70.3)	56.0 (46–70.0)	56.0 (43.0–72.0)	0.989
Significant tricuspid regurgitation^‡^, n (%)	138 (53.1)	97 (53.6)	41 (51.9)	0.667
TAPSE/sPAP ratio, mm/mmHg	0.32 (0.24–0.46)	0.33 (0.25–0.44)	0.32 (0.22–0.46)	0.460
TAPSE/sPAP ratio < 0.36, n (%)	113 (43.5)	75 (41.4)	38 (48.1)	0.595

NYHA indicates New York Heart Association; NT-proBNP, N-terminal prohormone of brain natriuretic peptide; ACE, angiotensin converting enzyme, E/e’, ratio of early transmitral blood velocity to early diastolic mitral annular velocity; TAPSE, tricuspid annular plane systolic excursion; sPAP, systolic pulmonary arterial pressure.

Values are given as median and interquartile range, or total numbers and percent.

^*^Estimated glomerular filtration rate < 60 mL/min/1.73 m^2^.

^†^Body mass index ≥ 30 kg/m^2^.

^‡^Moderate or severe tricuspid regurgitation.

Of the 260 HFpEF patients, 181 (69.6%) were female and 79 (30.4%) were male. Median age of female study participants was 73.0 years (IQR: 67.5–77.0) and 72.0 years (IQR: 66.0–77.0) in men (p = 0.237). Concomitant medications were equally distributed between men and women. Furthermore, men were more often current/former smokers (40.5% versus 23.8%, p = 0.009). Relevant gender differences were encountered with respect to cardiac and hemodynamic parameters. Compared to female patients, men had higher DPG (3.0 mmHg IQR: 0.0–6.0 versus 1.0 mmHg IQR: −2.0–4.5, p = 0.010), LV mass index (61.7 g/m^2^ IQR: 52.5–74.9 versus 53.6 g/m^2^ IQR: 44.3–63.2, p = 0.002), and RV end-diastolic volume indices (85.0 mL/m^2^ IQR: 66.8–96.1 versus 73.7 mL/m^2^ IQR: 62.0–84.5, p = 0.011), whereas LVEF (58.0% IQR: 53.0–66.0 versus 66.0% IQR: 55.5–74.0, p = 0.005), and RVEF were lower (50.0% IQR: 44.0–54.8 versus 55.0% IQR: 45.0–63.0, p = 0.006). No statistically significant differences with regard to prevalence of PH (75.9% versus 70.2%, p = 0.985), IpcPH (58.2% versus 60.2%, p = 0.121), CpcPH (17.7% versus 9.9%, p = 0.121) or TTE-assessed parameters of RV function could be detected between male and female HFpEF patients [TAPSE: 19.0 mm (IQR: 16.0–22.0) versus 19.0 mm (IQR: 15.8–23.3), p = 0.838; significant tricuspid regurgitation: 53.6% versus 51.9%, p = 0.667; TAPSE/sPAP: 0.33 mm/mmHg (IQR: 0.25–0.44) versus 0.32 mm/mmHg (IQR: 0.22–0.46), p = 0.460; TAPSE/sPAP < 0.36: 41.4% versus 48.1%, p = 0.595].

Overall, male study participants had lower exercise capacity (% of predicted 6-MWD: 66.7% IQR: 39.8–91.6 versus 78.1% IQR: 58.5–92.4, p = 0.036) and a higher burden of co-morbidities (atrial fibrillation: 68.4% versus 54.7%, p = 0.40; anemia: 73.4% versus 60.8%, p = 0.050; sleep apnea: 20.3% versus 5.0%, p < 0.001; COPD: 46.8% versus 27.1%, p = 0.002).

### Outcome according to gender

During a median follow-up period of 30.0months (IQR: 13.0–48.0), 24 (9.2%) patients reached the endpoint of cardiac death, 87 (33.5%) the combined endpoint of cardiac death or HF hospitalization and 45 (17.3%) reached the endpoint for all-cause death (Table 1). Men had higher rates of cardiac death (16.5% versus 6.1%, p = 0.008) and lower rates of non-cardiac death (2.5% versus 10.5%, p = 0.030) as compared to women (Table 1). Women more often died from infections (23.3% versus 0.0%, p = 0.042), whereas RHF (73.3% versus 36.7%, p = 0.020) and SCD (13.3% versus 0.0%, p = 0.041) were more frequent among men (Table 2).

**Table 2:** Modes of death according to gender.

Variable	Deaths total HFpEF cohort (n = 45)	Deaths female HFpEF cohort (n = 30)	Deaths male HFpEF cohort (n = 15)	*P* value
**Mode of death**				
**Right heart failure**, **n** (**%**)	22 (48.9)	11 (36.7)	11 (73.3)	**0**.**020**
**Sudden cardiac death**, **n** (**%**)	2 (4.4)	0 (0.0)	2 (13.3)	**0**.**041**
**Infection**, **n** (**%**)	7 (15.6)	7 (23.3)	0 (0.0)	**0**.**042**
Malignancy, n (%)	4 (8.9)	4 (13.3)	0 (0.0)	0.138
Other, n (%)*	10 (22.2)	8 (26.7)	2 (13.3)	0.310

*Other modes of death were: Stroke, periprocedural, ileus and unclear.

**Figure 1:**
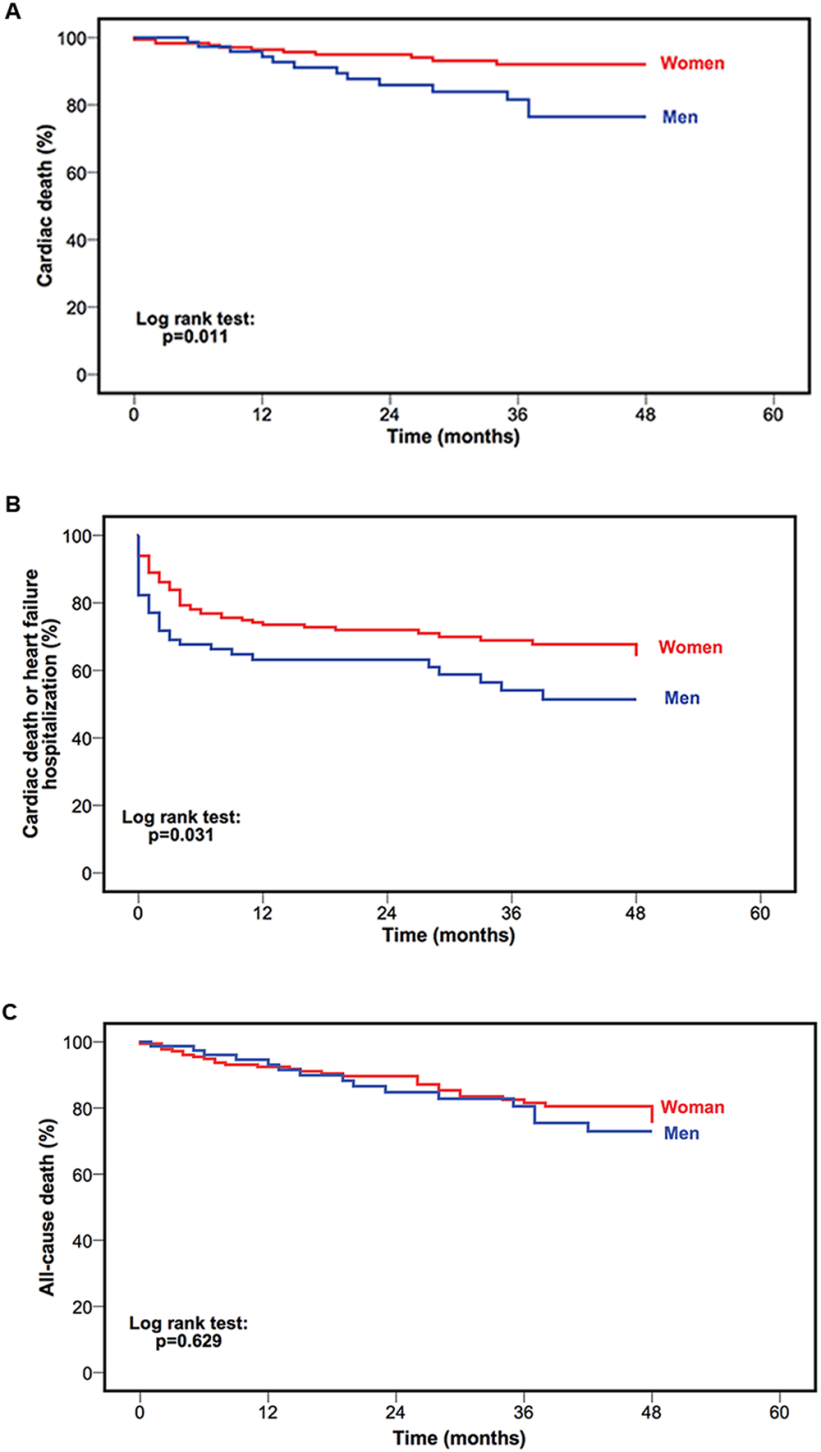
Kaplan-Meier curves stratified by gender for the endpoints cardiac death (**A**), cardiac death or heart failure hospitalization (**B**) and all-cause death (**C**) of patients with heart failure and preserved ejection fraction.

Uni- and multivariable Cox regression analyses for the HFpEF cohort are shown in Table 3, Supplemental Tables 1 and 2. In uni- and multivariable Cox regression as well as Kaplan-Meier analyses, male gender was independently associated with shorter time to cardiac death [hazard ratio (HR): 2.639, confidence interval (CI): 1.023–6.805, p = 0.045, Table 3, Fig. 1A]. In addition, male HFpEF patients reached the combined endpoint within a shorter time period as compared to their female counterparts (HR: 1.588, 95% CI: 1.029–2.450, p = 0.037, Supplemental Table 1, Fig. 1B). In the multivariable analysis including clinical parameters such as exercise capacity and NT-proBNP, male gender itself failed to predict event-free survival for the combined endpoint (Supplemental Table 1). There was no difference with regards to time to all-cause death between the two groups (HR: 1.164, 95% CI: 0.626–2.164, p = 0.631, Supplemental Table 2, Fig. 1C).

**Table 3:** Univariable and multivariable Cox regression analyses for the endpoint cardiac death.

Variable	Hazard ratio	95% Confidence interval	*P* value	Hazard ratio	95% Confidence interval	*P* value
	Univariable regression			Multivariable regression		
**Clinical parameters**						
Age, years	1.036	0.984–1.092	0.176			
**Male gender**	2.727	1.221–6.087	0.014	2.639	1.023–6.805	**0**.**045**
Body mass index, kg/m^2^	0.997	0.938–1.061	0.936			
**% of 6 minute walk distance predicted**, **%**	0.971	0.956–0.986	<0.001	0.981	0.964–0.997	**0**.**023**
NYHA functional class ≥ III	11.266	1.521–83.427	0.018			
**NT-proBNP**, pg/mL^*^	2.794	1.772–4.404	<0.001	2.554	1.525–4.277	<**0**.**001**
**Co-morbidities**						
Atrial fibrillation	2.675	0.999–7.163	0.050			
Non-significant coronary artery disease	0.847	0.316–2.268	0.740			
Hyperlipidemia	0.812	0.365–1.809	0.610			
Diabetes mellitus type II	1.195	0.531–2.690	0.668			
Anemia	2.114	0.789–5.664	0.137			
Sleep apnea	1.221	0.364–4.097	0.747			
Smoker (former or active)	1.434	0.627–3.278	0.393			
Chronic obstructive pulmonary disease^†^	1.382	0.918–3.113	0.435			
**Invasive hemodynamic parameters**						
Mean pulmonary arterial pressure, mmHg	1.054	1.018–1.092	**0**.**003**			
**Right atrial pressure**, **mmHg**	1.136	1.067–1.210	**<0**.**001**	1.130	1.059–1.205	**<0**.**001**
Pulmonary artery wedge pressure, mmHg	1.081	1.010–1.157	**0**.**026**			
Stroke volume index, mL/m^2^	0.989	0.949–1.029	0.578			
Cardiac index, L/min/m^2^	0.906	0.483–1.907	0.906			
Pulmonary vascular resistance, dyn·s·cm^−5^	1.003	1.001–1.005	**0**.**010**	1.002	1.000–1.005	**0**.**047**
Diastolic pressure gradient, mmHg	1.056	0.992–1.124	0.085			
**Cardiac magnetic resonance imaging parameters**						
Left atrial volume index, mL/m^2^	1.000	0.998–1.001	0.786			
Right atrial area index, mm/m^2^	1.104	1.022–1.192	0.012			
Left ventricular ejection fraction, %	0.967	0.924–1.011	0.137			
Left ventricular end-diastolic volume index, mL/m^2^	0.990	0.966–1.015	0.448			
Left ventricular mass index, g/m^2^	0.990	0.973–1.008	0.283			
**Right ventricular ejection fraction**, **%**	0.934	0.891–0.978	**0**.**004**	0.934	0.891–0.978	**0**.**004**
Right ventricular end-diastolic volume index, mL/m^2^	1.009	0.994–1.025	0.252			

NYHA indicates New York Heart Association; NT-proBNP, N-terminal prohormone of brain natriuretic peptide.

*NT-proBNP was graded into quintiles.

^†^Patients with severe chronic obstructive pulmonary disease (GOLD ≥ III) were excluded from the registry.

## Discussion

### Gender-related differences in heart failure with preserved ejection fraction

Epidemiological studies suggest that gender plays an important role in the development of HFpEF, which is reflected by a female predominance in this disease with a gender ratio of approximately 2:1^4,21^. In fact, a relatively large body of evidence suggests that women are more prone to develop a hypertrophied, stiff and non-dilated LV, which is pathognomonic for HFpEF^22,23^. Despite the fact that gender seems to play quite an important role for HFpEF evolution, only few studies have specifically investigated gender-related differences in pure HFpEF cohorts^6,7^.

Among the studies that have, it could be shown that women tend towards higher LVEF, worse diastolic function and less co-morbid conditions as compared to men^6,7^. However, despite their prognostic importance in HFpEF, PAP, RV function and exercise capacity have not been systematically assessed within the context of a HFpEF gender study^15,24–26^. In the present study, we have performed invasive hemodynamic assessment and CMR studies, which are the current gold standard for characterization of hemodynamics and cardiac function.

In line with previous data, in our cohort men were more often active or former smokers (40.5% versus 23.8%, p = 0.009)^6^. Men also had a higher burden of co-morbidities (atrial fibrillation: 68.4% versus 54.7%, p = 0.40; anemia: 73.4% versus 60.8%, p = 0.050; sleep apnea: 20.3% versus 5.0%, p < 0.001; COPD: 46.8% versus 27.1%, p = 0.002)^6^. CMR-measured LVEF was lower in male study participants (58.0% IQR: 53.0–66.0 versus 66.0% IQR: 55.5–74.0, p = 0.005), confirming results from an echocardiographic study by Gori *et al*.^7^. In addition, we found men to have a higher DPG (3.0 mmHg IQR: 0.0–6.0 versus 1.0 mmHg IQR: −2.0–4.5, p = 0.010), accompanied by RV enlargement (RV end-diastolic volume index: 85.0 mL/m^2^ IQR: 66.8–96.1 versus 73.7 mL/m^2^ IQR: 62.0–84.5, p = 0.011) and impaired function (RVEF: 50.0% IQR: 44.0–54.8 versus 55.0% IQR: 45.0–63.0, p = 0.006). These findings had clinical implications with more pronounced limitations in exercise capacity in men as compared to women (% of predicted 6-MWD: 66.7% IQR: 39.8–91.6 versus 78.1% IQR: 58.5–92.4, p = 0.036).

### Gender and outcome in heart failure with preserved ejection fraction

Gender strongly influences outcome in various cardiac conditions, such as myocardial infarction and HF with reduced EF^27^. Albeit, relatively little is known on the impact of gender on outcome in HFpEF, the existing literature suggests a worse clinical course for male HFpEF patients^6,28^.

In the present study three different endpoints were analyzed. In univariable survival analyses male gender was a predictor for the endpoint cardiac death (HR: 2.727 95% CI: 1.221–6.087 p = 0.014, Fig. 1A) and the combined endpoint of HF hospitalization or cardiac death (HR: 1.588, 95% CI: 1.029–2.450, p = 0.037, Fig. 1B), but not for all-cause death (HR: 1.164, 95% CI: 0.626–2.164, p = 0.631 Fig. 1C). In the multivariable model, which included parameters such as NT-proBNP and exercise capacity, male gender remained an independent predictor for cardiac death (HR: 2.639, 95% CI: 1.023–6.805, p = 0.045), but failed to do so for the combined endpoint.

Results from the I-Preserve trial, which included roughly 3000 HFpEF patients detected a worse outcome for male HFpEF patients, who had a higher risk for all-cause death^6^. This stands in contrast to the results from the present study, where gender was not predictive for this endpoint. This difference might be attributable to the fact that we have actively screened for and excluded significant CAD as well as cardiac amyloidosis. These conditions are associated with male gender, poor outcomes and could, if not ruled out, mimic HFpEF^9,29^. A recent publication by Hoeper and colleagues investigating 108 patients with HFpEF and subsequent pulmonary hypertension (PH) also found male gender to be an independent predictor of all-cause death^28^. However, Hoeper *et al*. only studied patients with PH-HFpEF and the present study included HFpEF patients with and without PH. This difference between the study populations could explain why male gender was not associated with all-cause mortality in our study.

### Gender and mode of death in heart failure with preserved ejection fraction

In the present study, mode of death (MOD) differed significantly between male and female HFpEF patients. Men almost exclusively died from RHF (73.3%) and SCD (13.3%), whereas MOD was more diverse among women (RHF: 36.7%, infection: 23.3%, malignancy: 13.3%). A recently published review investigated MOD of HFpEF patients in clinical trials as well as epidemiological studies^30^. By contrast to the existing literature, where the reported MOD was cardiovascular in more than two thirds of HFpEF patients, our results suggest a considerably higher rate of non-cardiovascular deaths^10,30^. One explanation for this discrepancy could be the enrichment of male participants in clinical HFpEF trials (Charm Preserved: 60%, Topcat: 52%, I-Preserve: 60%)^31–33^ compared to our all-comers registry (30.4%). Further explanations for these differences could be a lack of standardized definitions for MOD, different LVEF cutoffs across studies (Charm Preserved: > 40%, Topcat: ≥ 45%, I-Preserve: ≥ 45% versus ≥ 50% in the present study)^31–33^, younger patients populations in clinical trials (Charm Preserved: 67.2 ± 11.1 years, Topcat: 68.7 years IQR: 60.7–75.5, I-Preserve: 72.0 years ± 7.0 versus 73.0 years IQR: 67.0–77.0 in the present study)^31–33^, thus reducing the risk for non-cardiovascular deaths such as infections or malignancies.

### Limitations

One limitation of the present study is its single-center design. Even though a center-specific bias cannot be excluded, limiting data collection to one center has the advantage of a constant clinical work-up, constant clinical routine and constant follow-up. Compared to previous trials investigating gender differences, our study cohort is relatively small and the number of events is limited^6^. Furthermore, the duration of HF before patient enrollment has not been assessed. However, due to the systematic use of left- and right heart catheterization as well as CMR imaging we were able to study a very well-characterized pure HFpEF population.

## Conclusions

In this prospective study of a well-characterized HFpEF cohort we could demonstrate clear differences between male and female HFpEF patients. Men were more compromised with respect to clinical, functional and hemodynamic parameters, which seemed to explain worse cardiac outcome among them. Differences between genders in MOD suggest that men rather develop a right heart phenotype and die from HFpEF, whereas women are more likely to die with HFpEF^29^.

## Electronic supplementary material

Supplemental Tables 1 and 2
