# Supplementary material for: Gender-related differences in heart failure with preserved ejection fraction
Source: Sci Rep. 2018 Jan 18;8:1080. doi: 10.1038/s41598-018-19507-7 (PMC5773700; doi:10.1038/s41598-018-19507-7)
Supplement: Supplementary file 1 — Supplemental Tables 1 and 2 [file 41598_2018_19507_MOESM1_ESM.doc]

**Gender-related differences in heart failure with preserved ejection fraction**

**Running title:** Gender differences in HFpEF

Franz Duca, MD^1^; Caroline Zotter-Tufaro, PhD^1^; Andreas A. Kammerlander, MD^1^; Stefan Aschauer, MD^1^; Christina Binder, MD^1^; Julia Mascherbauer, MD^1^; Diana Bonderman, MD^1^

^1^Division of Cardiology, Department of Internal Medicine II, Medical University of Vienna, Vienna, Austria

**Supplemental table 1. Univariable and multivariable Cox regression analysis for the combined endpoint of heart failure hospitalization or cardiac death**

| **Variable** | **Hazard ratio** | | **95% Confidence interval** | | ***P* value** | **Hazard ratio** | **95% Confidence interval** | ***P* value** |
| --- | --- | --- | --- | --- | --- | --- | --- | --- |
|  | **Univariable regression** | | | | | **Multivariable regression** | | |
| **Clinical parameters** |  | |  | |  |  |  |  |
| Age, years | 1.008 | | 0.983 – 1.034 | | 0.531 |  |  |  |
| Male gender | 1.588 | | 1.029 – 2.450 | | **0.037** |  |  |  |
| Body mass index, kg/m^2^ | 1.023 | | 0.992 -1.055 | | 0.147 |  |  |  |
| **% of 6 minute walk distance predicted, %** | 0.979 | | 0.971 – 0.986 | | **<0.001** | 0.980 | 0.971 – 0.988 | **<0.001** |
| NYHA functional class ≥ III | 2.783 | | 1.595 – 4.857 | | **<0.001** |  |  |  |
| **NT-proBNP, pg/mL*** | 1.593 | | 1.350 - 1.879 | | **<0.001** | 1.491 | 1.251 – 1.777 | **<0.001** |
| **Co-morbidities** |  | |  | |  |  |  |  |
| **Atrial fibrillation** | 1.978 | | 1.242 – 3.148 | | **0.004** | 2.088 | 1.291 – 3.280 | **0.002** |
| Non-significant coronary artery disease | 1.000 | | 0.612 – 1.634 | | 0.999 |  |  |  |
| Hyperlipidemia | 0.797 | | 0.523 – 1.214 | | 0.290 |  |  |  |
| Diabetes mellitus type II | 1.528 | | 1.000 – 2.334 | | **0.050** |  |  |  |
| **Anemia** | 1.613 | | 1.008 – 2.582 | | **0.046** | 1.706 | 1.064 – 2.736 | **0.026** |
| Sleep apnea | 0.954 | | 0.461 – 1.975 | | 0.899 |  |  |  |
| Smoker (former or active) | 1.493 | | 0.964 – 2.312 | | 0.073 |  |  |  |
| Chronic obstructive pulmonary disease† | 1.410 | | 0.918 – 2.164 | | 0.117 |  |  |  |
| **Invasive hemodynamic parameters** |  | |  | |  |  |  |  |
| **Mean pulmonary arterial pressure, mmHg** | 1.050 | | 1.030 – 1.070 | | **<0.001** | 1.049 | 1.029 – 1.069 | **<0.001** |
| Right atrial pressure, mmHg | 1.082 | | 1.041 – 1.125 | | **<0.001** |  |  |  |
| Pulmonary artery wedge pressure, mmHg | 1.074 | | 1.035 – 1.115 | | **<0.001** |  |  |  |
| Stroke volume index, mL/m^2^ | 1.000 | | 0.978 – 1.021 | | 0.976 |  |  |  |
| Cardiac index, L/min/m^2^ | 0.829 | | 0.569 – 1.208 | | 0.330 |  |  |  |
| Pulmonary vascular resistance, dyn·s·cm^-5^ | 1.003 | | 1.002 – 1.005 | | **<0.001** |  |  |  |
| Diastolic pressure gradient, mmHg | 1.040 | | 1.000 – 1.081 | | 0.052 |  |  |  |
| **Cardiac magnetic resonance imaging parameters** | |  |  | |  |  |  |  |
| Left atrial volume index, mL/m^2^ | 1.000 | | | 0.999 – 1.001 | 0.588 |  |  |  |
| Right atrial area index, mm/m^2^ | 1.034 | | | 0.984 – 1.086 | 0.187 |  |  |  |
| Left ventricular ejection fraction, % | 0.998 | | | 0.974 – 1.023 | 0.902 |  |  |  |
| Left ventricular end-diastolic volume index, mL/m^2^ | 1.000 | | | 0.988 – 1.012 | 0.963 |  |  |  |
| Left ventricular mass index, g/m^2^ | 0.997 | | | 0.987 – 1.007 | 0.532 |  |  |  |
| **Right ventricular ejection fraction, %** | 0.962 | | | 0.940 – 0.984 | **0.001** | 0.962 | 0.940 – 0.984 | **0.001** |
| Right ventricular end-diastolic volume index, mL/m^2^ | 1.010 | | | 1.003 – 1.018 | **0.008** |  |  |  |

NYHA indicates New York Heart Association; NT-proBNP, N-terminal prohormone of brain natriuretic peptide.

*NT-proBNP was graded into quintiles for this analysis.

†Patients with severe chronic obstructive pulmonary disease (GOLD ≥III) were excluded from the registry.

**Supplemental table 2. Univariable and multivariable Cox regression analysis for the endpoint all-cause death**

| **Variable** | | | **Hazard ratio** | | | **95% Confidence interval** | | | ***P* value** | **Hazard ratio** | **95% Confidence interval** | ***P* value** |
| --- | --- | --- | --- | --- | --- | --- | --- | --- | --- | --- | --- | --- |
|  | | | **Univariable regression** | | | | | | | **Multivariable regression** | | |
| **Clinical parameters** | | |  | | |  | | |  |  |  |  |
| Age, years | | | 1.019 | | | 0.983 – 1.056 | | | 0.298 |  |  |  |
| Male gender | | | 1.164 | | | 0.626 – 2.164 | | | 0.631 |  |  |  |
| Body mass index, kg/m^2^ | | | 1.002 | | | 0.958 -1.048 | | | 0.942 |  |  |  |
| **% of 6 minute walk distance predicted, %** | | | 0.981 | | | 0.970 – 0.992 | | | **0.001** | 1.696 | 1.301 – 2.209 | **0.007** |
| NYHA functional class ≥ III | | | 4.977 | | | 1.783 – 13.897 | | | **0.002** |  |  |  |
| **NT-proBNP, pg/mL*** | | | 1.832 | | | 1.423 - 2.360 | | | **<0.001** | 0.984 | 0.973 – 0.996 | **<0.001** |
| **Co-morbidities** | |  | | | |  | | |  |  |  |  |
| Atrial fibrillation | | 1.413 | | | | 0.760 – 2.626 | | | 0.274 |  |  |  |
| Non-significant coronary artery disease | | 0.920 | | | | 0.455 – 1.858 | | | 0.816 |  |  |  |
| Hyperlipidemia | | 1.128 | | | | 0.624 – 2.038 | | | 0.691 |  |  |  |
| Diabetes mellitus type II | | 1.238 | | | | 0.685 – 2.237 | | | 0.480 |  |  |  |
| Anemia | | 1.245 | | | | 0.662 – 2.342 | | | 0.496 |  |  |  |
| Sleep apnea | | 1.019 | | | | 0.402 – 2.585 | | | 0.969 |  |  |  |
| **Smoker (former or active)** | | 1.866 | | | | 1.036 – 3.361 | | | **0.038** | 1.866 | 1.036 – 3.361 | **0.038** |
| Chronic obstructive pulmonary disease† | | 1.042 | | | | 0.566 – 1.920 | | | 0.895 |  |  |  |
| **Invasive hemodynamic parameters** | | |  | |  | | | |  |  |  |  |
| **Mean pulmonary arterial pressure, mmHg** | | | 1.046 | | 1.018 – 1.074 | | | | **0.001** | 1.046 | 1.018 – 1.074 | **0.001** |
| Right atrial pressure, mmHg | | | 1.074 | | 1.021 – 1.129 | | | | **0.005** |  |  |  |
| Pulmonary artery wedge pressure, mmHg | | | 1.078 | | 1.025 – 1.134 | | | | **0.004** |  |  |  |
| Stroke volume index, mL/m^2^ | | | 0.993 | | 0.964 – 1.022 | | | | 0.623 |  |  |  |
| Cardiac index, L/min/m^2^ | | | 0.934 | | 0.560 – 1.558 | | | | 0.794 |  |  |  |
| Pulmonary vascular resistance, dyn·s·cm^-5^ | | | 1.002 | | 1.001 – 1.004 | | | | **0.008** |  |  |  |
| Diastolic pressure gradient, mmHg | | | 1.034 | | 0.983 – 1.089 | | | | 0.198 |  |  |  |
| **Cardiac magnetic resonance imaging parameters** | | | |  | | | |  |  |  |  |  |
| Left atrial volume index, mL/m^2^ | 1.000 | | | | | | 0.999 – 1.001 | | 0.729 |  |  |  |
| Right atrial area index, mm/m^2^ | 1.059 | | | | | | 0.994 – 1.127 | | 0.074 |  |  |  |
| Left ventricular ejection fraction, % | 0.976 | | | | | | 0.945 – 1.007 | | 0.134 |  |  |  |
| Left ventricular end-diastolic volume index, mL/m^2^ | 1.004 | | | | | | 0.990 – 1.019 | | 0.558 |  |  |  |
| Left ventricular mass index, g/m^2^ | 0.995 | | | | | | 0.982 – 1.008 | | 0.457 |  |  |  |
| **Right ventricular ejection fraction, %** | 0.947 | | | | | | 0.916 – 0.979 | | **0.001** | 0.947 | 0.916 – 0.979 | **0.001** |
| Right ventricular end-diastolic volume index, mL/m^2^ | 1.004 | | | | | | 0.991 – 1.016 | | 0.563 |  |  |  |

NYHA indicates New York Heart Association; NT-proBNP, N-terminal prohormone of brain natriuretic peptide.

*NT-proBNP was graded into quintiles for this analysis.

†Patients with severe chronic obstructive pulmonary disease (GOLD ≥III) were excluded from the registry.
